# *EHP* Paper of the Year, 2011

**DOI:** 10.1289/ehp.1104461

**Published:** 2011-11-01

**Authors:** Hugh A. Tilson

**Affiliations:** Editor-in-Chief, *EHP*, E-mail: tilsonha@niehs.nih.gov

In 2008, *Environmental Health Perspectives* (*EHP*) established the Paper of the Year Award as a means of recognizing high-impact papers published in the journal (Tilson 2008). Originally, the Paper of the Year was selected on the basis of citations received over the preceding 60 months. Earlier this year *EHP* announced that it would be recognizing two papers each year (Tilson 2011). The *EHP* Classic Paper of the year will be the research article, commentary, or review article that is the most highly cited paper over the preceding 60 months. The winner of the 2011 *EHP* Classic Paper of the Year was “Maternal Genistein Alters Coat Color and Protects A*^vy^* Mouse Offspring from Obesity by Modifying the Fetal Epigenome” by Dana C. Dolinoy, Jennifer R. Weidman, Robert A. Waterland, and Randy Jirtle. The second paper to be recognized each year will be the *EHP* Paper of the Year. This award will recognize a highly cited paper published during the preceding year. Both awards are subject to the approval of the *EHP* Board of Associate Editors.

In this issue, *EHP* announces that the Paper of the Year for 2011 is “Global Estimates of Ambient Fine Particulate Matter Concentrations from Satellite-Based Aerosol Optical Depth: Development and Application” by Aaron van Donkelaar, Randall V. Martin, Michael Brauer, Ralph Kahn, Robert Levy, Carolyn Verduzco, and Paul J. Villeneuve. This paper (van Donkelaar et al. 2010) was published in the June 2010 issue of the journal.

At the time the paper was written, it was clear that chronic exposure to fine particulate matter (< 2.5 µm in diameter; PM_2.5_) could harm human health by producing morbidity and mortality. van Donkelaar et al. (2010) recognized that the sparseness of monitoring data, especially in developing countries, had hindered epidemiologic and health impact studies of PM_2.5_. They noted that satellite remote sensing offers unparalleled global coverage of aerosol optical depth (AOD), a measure of aerosol over the entire atmospheric column, and that the relationship between AOD and near-surface PM_2.5_ varies in both space and time. van Donkelaar et al. (2010) related AOD to near-surface PM_2.5_ by using a chemical transport model that predicts the state of the atmosphere from meteorologic data sets, emission inventories, and equations that represent the physical and chemical evolution of atmospheric constituents.

van Donkelaar et al. (2010) combined AODs from two NASA (National Aeronautics and Space Administration) satellite instruments [Moderate Resolution Imaging Spectroradiometer (MODIS) and Multi-angle Imaging Spectroradiometer (MISR)] with the Goddard Earth Orbiting System chemical transport model to produce global long-term average (2001–2006) PM_2.5_ estimates at approximately 10 km × 10 km horizontal resolution. They found significant agreement with ground-based PM_2.5_ monitors, where present, both in North America and around the world.

van Donkelaar et al. (2010) provided an observationally based data set to assess the impact of ambient PM_2.5_ exposure globally. The authors estimated that the World Health Organization (WHO) Air Quality Guideline of 10 μg/m^3^ is exceeded for 80% of the world’s population. Over eastern Asia, half the population has annual ambient mean PM_2.5_ exposures in excess of the WHO PM_2.5_ interim target of 35 μg/m^3^, and in eastern China annual mean levels exceed 80 μg/m^3^.

*EHP* congratulates van Donkelaar and colleagues for their contribution to the environmental health science literature. Their findings have broad implications for the global environmental health and risk assessment communities.
